# Beyond VoF: alternative OpenFOAM solvers for numerical wave tanks

**DOI:** 10.1007/s40722-020-00173-9

**Published:** 2020-09-29

**Authors:** Pál Schmitt, Christian Windt, Josh Davidson, John V. Ringwood, Trevor Whittaker

**Affiliations:** 1 Marine Research Group, Queen’s University Belfast, Portaferry, BT11 1PF Northern Ireland UK; 2 Centre for Ocean Energy Research, Maynooth University, Co. Kildare, Ireland; 3 Department of Fluid Mechanics, Faculty of Mechanical Engineering, Budapest University of Technology and Economics, Budapest, Hungary

## Abstract

The vast majority of numerical wave tank applications are solved using finite volume-based, volume of fluid methods. One popular numerical modelling framework is *OpenFOAM* and its two phase solvers, *interFoam* and *interIsoFoam*, enabling the simulation of a broad range of marine hydrodynamic phenomena. However, in many applications, certain aspects of the entire set of possible hydrodynamic phenomena are not of interest and the reduced complexity could allow the use of simpler, more computationally efficient solvers. One barrier for the application of such alternative solvers is the lack of suitable wavemaking and absorption capabilities, which this paper aims to address. A wavemaking and absorption methodology is presented, which can be applied to different solvers using the same fundamental concept. The implementation is presented for *interFoam* and *interIsoFoam*, as well as two other solvers whose use as numerical wave tanks has not previously been reported in the literature, *shallowWaterFoam* and *potentialFreeSurfaceFoam*. Parameter studies are performed to guide the user in the use of the methods. Example applications for two industrially relevant test cases are demonstrated; a multi-frequency wave packet focused at one position over flat bottom and regular waves propagating over a submerged shoal. All solvers yielded useful results, but some complex wave transformations in the shoal case were only resolved by the VoF methods. Alternative methods beyond the already well established VoF methods seem worth considering because potential for significant reductions in computational effort exist.

## Introduction

Numerical simulations are an integral part of offshore and coastal engineering. Despite the often considerable computational cost, the flexibility of numerical tools, allowing investigation of different experimental designs and arbitrary tank layouts, with the ability to passively measure any variable in all locations throughout the tank, have seen an increase in the development of so called numerical wave tanks (NWTs) (Kim et al. 1999; Schmitt et al. 2012; Kim et al. 2016). NWTs have been demonstrated, for many applications, to yield results within the same level of accuracy as experimental tests, providing a reliable virtual test-bed for marine engineering, at significantly reduced cost compared to large scale experimental wave tank tests.

A major catalyst for the increasing usage of NWTs has been the availability of open-source software, eliminating license costs and providing users with ready made solvers and toolboxes for NWT applications. A popular open-source tool among researchers and industry, across many areas of offshore, coastal, and marine engineering, is *OpenFOAM* (Weller et al. 1998). For example, in a recent review of CFD-based NWTs for wave energy applications by Windt et al. (2018), *OpenFOAM* was the most highly used software, appearing in nearly twice as many publications as the second most popularly used software, Ansys Fluent (ANSYS 2019).

**Fig. 1:**
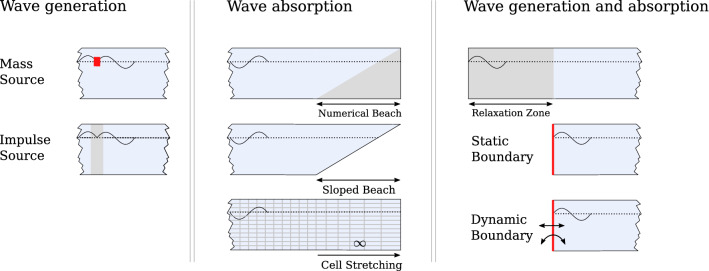
Depiction of the different wavemaker methods (adapted from Windt et al. (2009b))

The development of *OpenFOAM* NWTs is most commonly based on the volume of fluid (VoF) approach, implemented in the *interFoam* and the more recently developed *interIsoFoam* solvers (see Sect. 2.1). VoF methods can resolve breaking waves and other similarly complex flow features, and are thus the most universally applicable methods. However, VoF methods also have a number of drawbacks, which mainly lead to an increase in computational demand and requirement of:

- very high mesh resolution around the free surface.
- additional field variables and equations for species transport.
- small time steps due to unphysical high air velocities at the free surface, caused by the momentum transfer from the dense water to much lighter air.
- additional interpolation to retrieve surface elevation in post processing.

In many applications, breaking waves are not relevant and the reduced complexity could allow the use of potentially less computationally demanding methods. Two candidate methods are available in *OpenFOAM*:

1. Surface tracking: this method simply requires one additional parameter, which only needs to be evaluated on the water surface and not over the entire domain. The surface tracking method is implemented in the *potentialFreeSurfaceFoam* solver.
2. Shallow water equations: for the case of shallow water waves, the equations describing continuity and conservation of impulse can be further simplified into a two-dimensional (2-D) representation (Barré de Saint-Venant 1871). The wave action is assumed constant over the water depth, thus, drastically simplifying the system of equations and increasing the computational speed. The shallow water equations are implemented in the *shallowWaterFoam* solver.

The authors are not aware of any previous publication applying either of these solvers to implement a NWT. One possible barrier inhibiting the use of *potentialFreeSurfaceFoam* and *shallowWaterFoam* solvers is the lack of suitable wave making and absorption capabilities.

### Wavemakers

There are a number of methods for creating waves, as depicted in Fig. 1. Wave creation can be achieved with the mass source and impulse methods. Waves can be absorbed, and reflections mitigated, by a numerical beach, sloped beach, or cell stretching methods. The relaxation zone, static boundary, and dynamic boundary methods can deliver both wave creation and absorption. For more details of these different methods, see the review by Windt et al. (2009b).

Due to the popularity of the VoF method, there have been a number of wavemaker toolboxes implemented for the *interFoam* solver. The *waves2Foam* toolbox (Jacobsen et al. 2012) implements the relaxation method while *IHFoam* and *olaFlow* toolboxes (Higuera et al. 2013) implement both static and dynamic boundary methods. *OpenFOAM v7* includes a static boundary wavemaker implemented for the *interFoam* solver.

**Fig. 2:**
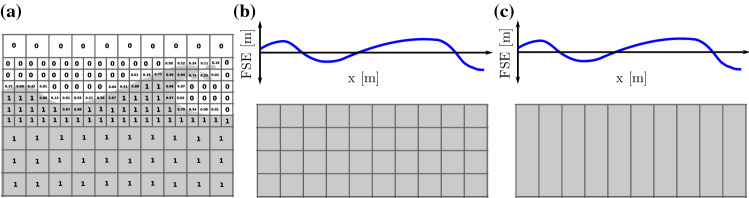
Depiction of methods to capture the free surface. **a** The surface capturing method, where the VoF indicates the fraction of water present in each cell ranging from pure air (0) to pure water (1). **b** The surface tracking method and the **c** shallow water approach, where the free surface elevation is a single valued function of position in the NWT

However, these wavemaker toolboxes are not available for the *potentialFreeSurfaceFoam* and *shallowWaterFoam* solvers. Due to the differences between the various solvers, the implementation of these wavemaker toolboxes would require significant adaptions to be compatible with the *potentialFreeSurfaceFoam* and *shallowWaterFoam* solvers. An easy alternative is the impulse source wavemaker, which requires only minor changes to the flow solver and is, thus, easily implemented (Schmitt et al. 2019; Feng and Wu 2019; Schmitt 2017).

### Objectives

This paper presents the implementation of new types of NWTs in OpenFOAM, based on enhancing the surface tracking and shallow water equation solvers with the impulse source wavemaker recently presented for the VoF method (Schmitt et al. 2019). The paper aims to demonstrate the ability of the impulse source wavemaker to be readily applied to different solvers without requiring extensive changes or tailoring. The underlying objective is to provide a diversity of solvers with the functionality required for NWT simulations, to broaden the range of available ocean engineering methods in OpenFOAM, and to foster discussion and developments beyond the already well established VoF methods.

### Outline

The paper is organized as follows: Sect. 2 presents the theoretical background of surface capturing, surface tracking, and shallow water equations. Section 3 describes the implementation of the wavemaker and numerical beach to the solvers. Two test cases are then presented, which are selected to demonstrate a useful application of all solvers at the same time, allowing a fair comparison, while highlighting any differences in the performance of the solvers. The test case in Sect. 4 considers a multi-frequency wave packet focused at one position and the test case in Sect. 5 presents regular wave propagation over a submerged shoal. Finally, conclusions are drawn in Sect. 6.

## Theoretical background

The dynamics of fluids in the ocean can be governed by the Navier–Stokes equations, describing the conservation of momentum and mass through the impulse and continuity equations:

1 $$\begin{aligned}&\frac{\partial (\rho \mathbf {U})}{\partial t}+ \nabla \cdot (\rho \mathbf {U}\mathbf {U}) = -\nabla p +\nabla \cdot \mathbf {T} + \rho \mathbf {F}_\mathrm{b} \end{aligned}$$

2 $$\begin{aligned}&\frac{\partial \,\rho }{\partial \,t}+\nabla \cdot \rho \mathbf {U} = 0 \end{aligned}$$

where *t* denotes time, $\mathbf {U}$ is the fluid velocity, *p* is the fluid pressure, $\rho$ is the fluid density, and $\mathbf {F}_\mathrm{b}$ is any external force such as gravity. The viscous stress tensor follows $\mathbf {T}=\mu \nabla ^2\mathbf {U}+\frac{1}{3}\nabla (\nabla \cdot \mathbf {U})$, with the dynamic viscosity $\mu$. $\nabla$ denotes the gradient operator, $\nabla \cdot$ denotes the divergence and $\mathbf {U}\mathbf {U}$ is the tensor product of two vectors.

*OpenFOAM* solves the above equations using a Eulerian finite volume formulation (Ferziger and Peric 2002). A main challenge with NWTs is the accurate modelling of the free surface. Three methods for modelling the free surface, the surface capturing/VoF method, the surface tracing method, and the shallow water approach, are depicted in Fig. 2 and outlined in Sects. 2.1, 2.2, and 2.3, respectively. Figure 2 also highlights the requirement of VoF methods to measure the free surface elevation, whereas in the other methods, free surface elevation is available as a variable.

### Surface capturing

The most commonly used method, and the only one able to simulate breaking or overtopping waves, is the VoF approach (Hirt and Nichols 1981). The volume fraction of water, $\alpha$, is captured by solving the following additional equations

3 $$\begin{aligned}&\frac{\partial \,\alpha }{\partial \,t}+\nabla \cdot \left( \mathbf {U}\alpha \right) =0 \end{aligned}$$

4 $$\begin{aligned}&\varPhi = \alpha \varPhi _{\text {water}}+(1-\alpha )\varPhi _{\text {air}}\,, \end{aligned}$$

where $\varPhi$ is a specific fluid quantity like density or viscosity.

Two different VoF solvers are available in *OpenFOAM*, namely *interFoam* and *interIsoFoam*. The difference between these two solvers is the algorithm used for maintaining a sharp interface. The *interFoam* solver employs an artificial compression velocity term (Deshpande et al. 2012), whereas *interIsoFoam* splits cells across the interface position (Roenby et al. 2016). An in-depth evaluation of *interFoam*, for a variety of multiphase flow applications, is presented by Deshpande et al. (2012).

For the case of wave propagation, *interIsoFoam* has been demonstrated to be of comparable accuracy to *interFoam*, while maintaining a sharper surface (Larsen et al. 2019). Indeed, while VoF methods have traditionally been characterised by excessive wave dissipation, the *isoAdvector* algorithm in *interIsoFoam* has been shown to significantly reduce this dissipation, for example to only 3.8% over 8 wavelengths for the case presented by Vukčević et al. (2018). Considerable improvements have also recently been achieved for the turbulence modelling around the free surface (Larsen and Fuhrman 2018).

### Surface tracking

The *potentialfreeSurfaceFoam* solver is an extension of the commonly used single-phase *pimpleFoam* solver for single phase flow. It employs a boundary condition called *waveSurfacePressure* which implements a surface tracking algorithm. The variable $\zeta$ stands for surface deformation from still-water level and can be used to visualise surface elevation on the surface boundary patch. The change in surface elevation $\delta \zeta$ for time step $\delta t$^1^ is updated from the volume flux $\phi$, the normalised face normal $\mathbf {n}$ and face area *A* according to:

5 $$\begin{aligned} \delta \zeta =\delta t \mathbf {n} \phi /A \end{aligned}$$

The authors are only aware of one research publication providing details on the actual solver (Paredes et al. 2012) and a single published application of the solver involving cross-flow tidal turbines operating in shallow water (Feinberg 2019). It should be noted that other surface tracking algorithms have been demonstrated to work successfully [for example see (Vukcevic et al. 2017)]; however, the work in the present paper is focused on solvers available in the main *OpenFOAM* branch.

### Shallow water

Simplified versions of the impulse and continuity equations can be derived for shallow water cases, where the wave action extends across the entire water depth, *h*, and can be represented at each position by a single depth averaged horizontal velocity value $\mathbf {u}$ (Barré de Saint-Venant 1871). In the resulting equations

6 $$\begin{aligned} \frac{\partial }{\partial t}(h \mathbf {u})+\nabla \cdot (h \mathbf {u}^T \mathbf {u})+ f \cdot h \mathbf {u}&= - | g | h \nabla (h+h_0) \end{aligned}$$

7 $$\begin{aligned} \frac{\partial }{\partial t} (h+h_0)+\nabla \cdot (h\mathbf {u})&=0\,, \end{aligned}$$

*f* denotes the Coriolis force, required for large scale ocean simulations, and $h_0$ is the deviation from the mean water-depth. The surface elevation is solved for on 2-D horizontal meshes and is directly available as a simulation variable.

## Wavemaker and numerical beach

The wave generation and absorption capability is added to all solvers in virtually identical ways, following the concept as described by Schmitt et al. (2019), which simply requires the addition of impulse terms to the Navier–Stokes equations without necessitating any other changes to the solvers or numerical solution approach.

### *interFoam* and *interIsoFoam*

The implementation of wavemakers and numerical beaches for both solvers is identical and a direct extension of the recently presented work on wavemakers by Schmitt et al. (2019). To implement the impulse sources for wave generation, as well as a numerical beach for wave absorption, two terms are added to Eq. (1), in the VoF solvers:

- $r_w \rho \mathbf {A}_{wm}$ : This is the source term used for wave generation, where $r_w$ is a binary scalar variable that defines the wavemaker region and $\mathbf {A}_{wm}$ is the acceleration input to the wavemaker at each cell centre within $r_w=1$. This term is based on the implementation of an internal wavemaker (Schmitt et al. 2019).
- $S \mathbf {n}_z \rho \mathbf {U}$ : This describes a dissipation term used to implement a numerical beach, where the variable *s*, with unit $[\hbox {s}^{-1}]$, controls the strength of the dissipation (Schmitt and Elsässer 2015). Compared to the implementation in Schmitt et al. (2019) and Schmitt and Elsässer (2015), in this study the beach only acts in the vertical, z-direction.

The extension of Eq. (1), by the additional terms for wave generation and absorption, yields the enhanced impulse equation:

8 $$\begin{aligned} \frac{\partial (\rho \mathbf {U})}{\partial t}+ \nabla \cdot (\rho \mathbf {U}\mathbf {U})= & {} -\nabla p +\nabla \cdot \mathbf {T} + \rho \mathbf {F}_\mathrm{b}\nonumber \\&+ r_w \rho \mathbf {A}_{wm} + S \mathbf {n}_z \rho \mathbf {U}. \end{aligned}$$

Modifying this equation in the respective solvers yields the *interFoamsrc* and *interIsoFoamsrc* solvers utilised in this paper. Note that, in the following, the added acronym *src* indicates the implementation of the impulse source wavemaker to the specific solver.

**Fig. 3:**
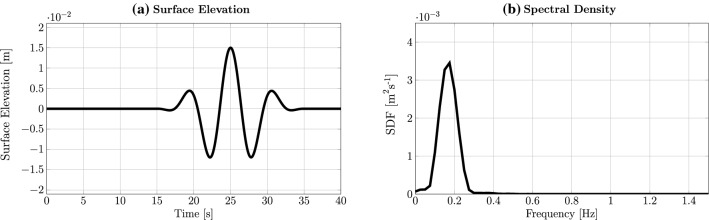
Unidirectional multi-frequency focused wave group: surface elevation (**a**) and spectral density (**b**)

### *potentialFreeSurfaceFoam*

Because *potentialFreeSurfaceFoam* solves a single phase, incompressible flow the density is constant and the impulse equation (1) can be simplified:

9 $$\begin{aligned} \frac{\partial (\mathbf {U})}{\partial t}+ \nabla \cdot ( \mathbf {U}\mathbf {U})= & {} -\frac{\nabla p}{\rho } +\frac{\nabla \cdot \mathbf {T}}{\rho }\nonumber \\&+ \mathbf {F}_\mathrm{b} + r_w \mathbf {A}_{wm} + S \mathbf {n}_z \mathbf {U}. \end{aligned}$$

The source terms for wave generation and the numerical beach described above in Sect. 3.1 need to be adjusted accordingly by simply dividing by density. The variables $\mathbf {A}_{wm}$ and *S* maintain the same units and meaning as introduced earlier for the VoF solvers. However, as shown later, the numerical values differ.

The resulting enhanced version of *potentialFreeSurfaceFoam* is called *potentialFreeSurfaceFoamsrc* in this paper

### *shallowWaterFoam*

The extension of the shallow water equation (7) is similar to the extension of Eqs. (8) and (9) discussed above; however, the units change since the solver solves for depth-averaged vertical velocity.

- $r_w \mathbf {a}_{wm}$ : this is the source term used for wave generation, where $r_w$ is a binary scalar variable that defines the wavemaker region and $\mathbf {a}_{wm}$ is the the depth integrated acceleration input to the wavemaker within $r_w=1$, with the same units as $h \mathbf {u}$.
- $s h \mathbf {u}$ : the dissipation term used to create a numerical beach, where the variable *s*, with unit $[\hbox {s}^{1}]$, controls the strength of the dissipation (Schmitt and Elsässer 2015).

**Fig. 4:**
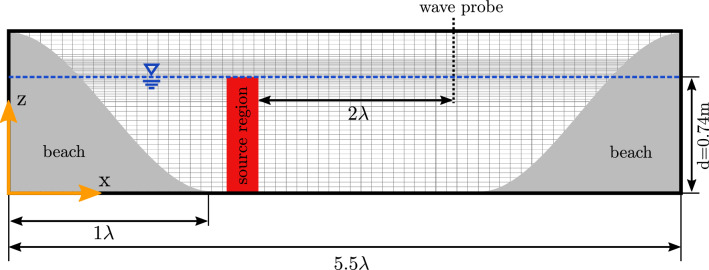
Schematic of the numerical wave tank

**Fig. 5:**
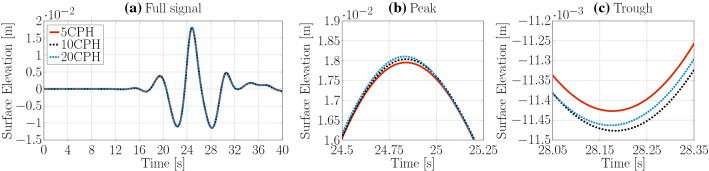
Spatial convergence study *interFoamsrc*

**Table 1:** Spatial and temporal convergence *interFoamsrc*

Spatial					
$\hbox {Co}_{\text {max}}$	$\varDelta \,z=\hbox {5\,CPH}$	$\varDelta \,z=\hbox {10\,CPH}$	$\varDelta \,z=\hbox {20\,CPH}$	Convergence Type	$\bar{U}$
0.2	0.0294	0.0295	0.0296	Monotone	$0.18\%$

Temporal					
$\varDelta \,z$	$\hbox {Co}_{\text {max}}=0.4$	$\hbox {Co}_{\text {max}}=0.2$	$\hbox {Co}_{\text {max}}=0.1$	Convergence Type	$\bar{U}$
10 CPH	0.0294	0.0295	0.0295	Monotone	$1.12\%$

The enhanced shallow water impulse equation used in the new *shallowWaterFoamsrc* solver is thus

10 $$\begin{aligned} \frac{\partial }{\partial t}(h \mathbf {u})+\nabla \cdot (h \mathbf {u}^T \mathbf {u})+ f \cdot h \mathbf {u}&= - | \mathbf (g) | h \nabla (h+h_0)\nonumber \\&\quad + r_w \mathbf {a}_{wm} - s h \mathbf {u} \end{aligned}$$

Features of the four modified solvers are demonstrated in the following sections for two test cases:

- A multi-frequency wave packet focused at one position (see Sect. 4).
- Regular wave propagation over a submerged shoal (see Sect. 5).

## Test case 1: multi-frequency wave packet

A unidirectional multi-frequency focused wave group, following the NewWave formulation (Ning et al. 2009), is considered in this section. The wave characteristics are: peak amplitude $A_0=0.015\,\hbox {m}$, peak period $T_\mathrm{p}=6.0\,\hbox {s}$, and a peak wave length $\lambda _\mathrm{p}=15.9\,\hbox {m}$, resulting in shallow water conditions ($\frac{d}{\lambda } =0.047$) at a water depth of $d=0.74\,\hbox {m}$. For brevity, the peak wave length $\lambda _\mathrm{p}$ and peak period $T_\mathrm{p}$ will henceforth be referred to simply as wave length $\lambda$ and wave period *T*. The surface elevation and spectral density of the focused wave group are plotted in Fig. 3a, b, respectively. The iterative calibration procedure applied by Schmitt et al. (2019) will be employed here to generate the desired focused wave group. A generic schematic of the NWT is depicted in Fig. 4. Note that the exact layout of the NWT varies, dependent on the employed solver.

As part of the NWT setup, temporal and spatial convergence studies are required to ensure appropriate levels of temporal and spatial discretisation, as detailed in Sect. 4.1. Furthermore, given the nature of the numerical wavemaker and beach, parametric studies on the numerical beach length and damping factor, as well as the source geometry, are required, as presented in Sects. 4.2 and 4.3, respectively. The application of the four different solvers is then presented in Sect. 4.4.

### Convergence study

Spatial and temporal convergence studies are performed independently for each of the modified solvers. To quantify the convergence behaviour, the method proposed by Roache (1997), Stern et al. (2001), and Vukcevic (2016) is applied, with specific focus on the discretisation uncertainty. The peak-to-trough wave height of the focused wave group, for three different discretisation sizes, is employed as metric in the convergence study.

**Fig. 6:**
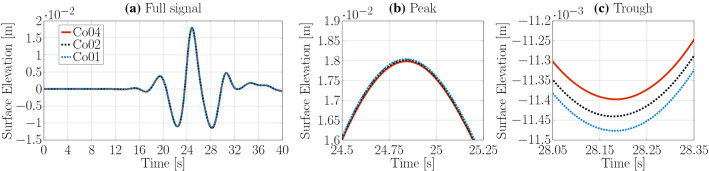
Temporal convergence study *interFoamsrc*

**Fig. 7:**
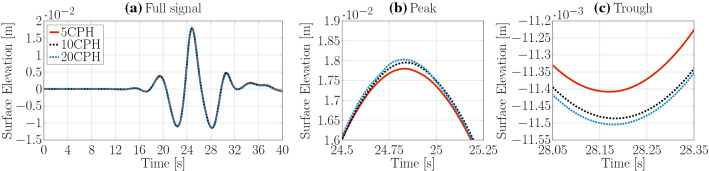
Spatial convergence study *interIsoFoamsrc*

**Fig. 8:**
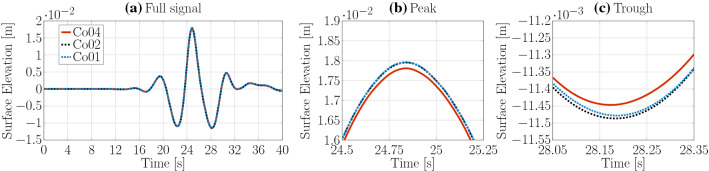
Temporal convergence study *interIsoFoamsrc*

**Table 2:** Spatial and temporal convergence *interIsoFoamsrc*

Spatial					
$\hbox {Co}_{\text {max}}$	$\varDelta \,z=\hbox {5\,CPH}$	$\varDelta \,z=\hbox {10\,CPH}$	$\varDelta \,z=\hbox {20\,CPH}$	Convergence type	$\bar{U}$
0.2	0.0292	0.0294	0.0295	Monotone	$0.31\%$

Temporal					
$\varDelta \,z$	$\hbox {Co}_{\text {max}}=0.4$	$\hbox {Co}_{\text {max}}=0.2$	$\hbox {Co}_{\text {max}}=0.1$	Convergence type	$\bar{U}$
10 CPH	0.0293	0.0294	0.0294	Oscillatory	$0.47\%$

#### *interFoamsrc*

For VoF methods, it is common practice to perform the spatial convergence study on the required grid size in the free surface interface region (Windt et al. 2018). The vertical cell size is then normalised by the wave height. Here, three different discretisation sizes are considered: 5, 10, and 20 cells per wave height (CPH). The horizontal cell size is selected based on a trade-off between maintaining the ideal aspect ratio of 1 (Ferziger and Peric 2002) and reducing the overall cell count in the domain to allow faster computation. In the bulk of the domain the aspect ratio, of horizontal to vertical cell size, is set to 2 and in the interface region the vertical mesh resolution is refined by one level, resulting in an aspect ratio of 4.

For the three grid resolutions, Fig. 5a–c show the complete wave signal, as well as a zoom on the highest peak and lowest trough, respectively. Only marginal differences can be observed in plotted time traces. Results for the different grid resolutions are listed in Table 1. Note that, for the spatial convergence study, a variable time step size with a Courant number condition $\text {Co}_{\text {max}}=0.2$ is used. The results indicate a monotonically converged solution for a grid resolution of 10 CPH with a discretisation uncertainty $\bar{U}=0.18\%$.

Similarly, three different maximum Courant numbers, i.e. $\text {Co}_{\text {max}}=0.1$, 0.2, 0.4, have been considered for the temporal convergence study. The resulting time traces are shown in Fig. 6 and the peak-to-trough wave heights are listed in Table 1. As for the spatial convergence study, only marginal differences at the wave peak and trough can be observed and monotonically converged results are achieved with a $\text {Co}_{\text {max}}=0.2$, resulting in a discretisation uncertainty of $\bar{U}=1.12\%$.

**Fig. 9:**
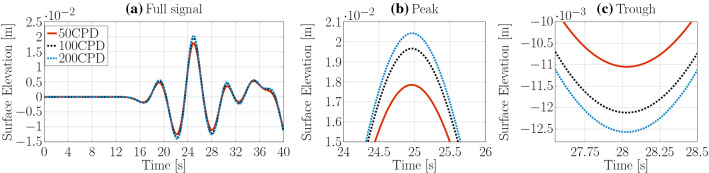
Spatial convergence study *potentialFreeSurfaceFoamsrc*

**Table 3:** Spatial and temporal convergence *potentialFreeSurfaceFoamsrc*

Spatial					
$\hbox {Co}_{\text {max}}$	50 CPD	100 CPD	200 CPD	Convergence type	$\bar{U}$
0.05	0.0303	0.0332	0.0345	Monotone	$3.78\%$

Temporal					
$\varDelta \,z$	$\hbox {Co}_{\text {max}}=0.1$	$\hbox {Co}_{\text {max}}=0.05$	$\hbox {Co}_{\text {max}}=0.025$	Convergence type	$\bar{U}$
100 CPD	0.0312	0.0332	0.0344	Monotone	$5.81\%$

**Fig. 10:**
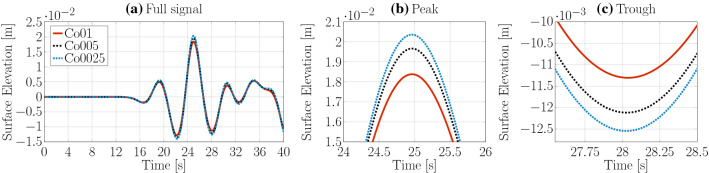
Temporal convergence study *potentialFreeSurfaceFoamsrc*

#### *interIsoFoamsrc*

Identical spatial and temporal discretisation sizes, as for the *interFoamsrc* solver, are considered for the convergence study of the *interIsoFoamsrc* solver. The resulting surface elevation time traces are plotted in Figs. 7 and 8 for the spatial and temporal convergence study, respectively. The peak-to-trough wave heights, as well as the convergence characteristics, are listed in Table 2. Converged results are achieved with a grid resolution of 10 CPH ($\bar{U}=0.31\%$) and a maximum Co number of 0.2 ($\bar{U}=0.47\%$). Note that the temporal convergence study indicates oscillatory convergence, while for *interFoamsrc* monotonic convergence was achieved. However, with a discretisation uncertainty of 0.47%, good convergence behaviour is ensured.

#### *potentialFreeSurfaceFoamsrc*

While the spatial discretisation size is expressed in terms of CPH in the interface region for the VoF based solvers, the *potentialFreeSurfaceFoamsrc* solver does not directly resolve the free surface in the mesh and, thus, discretisation sizes are expressed in terms of cells per water depth (CPD). Three different mesh resolutions are considered for the spatial convergence study: 50, 100, and 200 CPD. The mesh is uniform in vertical direction and the maximum cell aspect ratio is set to 2 (since no additional refinement layer is required at the free surface interface, like for the VoF solvers which had a maximum aspect ratio of 4). Again, variable time stepping is employed with $\text {Co}_{\text {max}}=0.05$.

Time traces of the free surface elevation, for the three different grid resolutions, are plotted in Fig. 9. Compared to the results from the two VoF based solvers, larger differences can be observed between the different grid resolutions. This is reflected in the peak-to-trough wave heights, as well as the convergence behaviour (see Table 3). While monotonic convergence is still achieved with 100CPD, the discretisation uncertainty $\bar{U}$ is relatively large (3.78%), compared to the VoF based solvers.

**Fig. 11:**
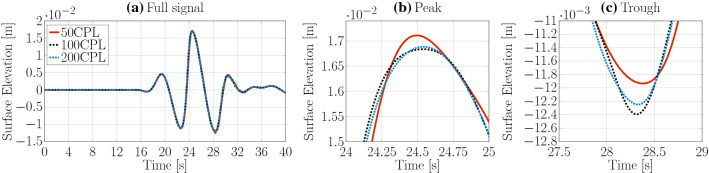
Spatial convergence study *shallowWaterFoamsrc*

**Table 4:** Spatial and temporal convergence *shallowWaterFoamsrc*

Spatial					
$\frac{\text {T}}{\text {d}t}$	50 CPL	100 CPL	200 CPL	Convergence type	$\bar{U}$
100	0.0290	0.0292	0.0291	Oscillatory	$0.48\%$

Temporal					
$\varDelta \,x$	$\text {d}t=\frac{\text {T}}{100}$	$\text {d}t=\frac{\text {T}}{200}$	$\text {d}t=\frac{\text {T}}{400}$	Convergence type	$\bar{U}$
100CPL	0.0292	0.0287	0.0286	Monotone	$0.31\%$

**Fig. 12:**
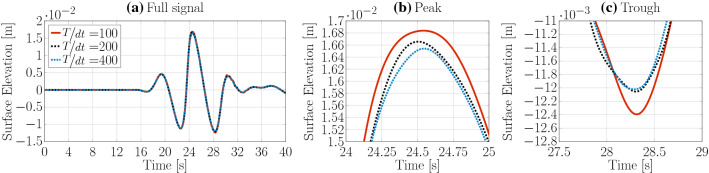
Temporal convergence study *shallowWaterFoamsrc*

For the temporal convergence study, three different maximum Co numbers (0.025, 0.05, 0.1) are tested. Time traces of the free surface elevation and the convergence characteristics are plotted in Fig. 10 and listed in Table 3. Monotonically converging results are found with $\text {Co}_{\text {max}}=0.05$ with a relatively large discretisation uncertainty of 5.81% compared to the VoF based solvers. This is noteworthy, since the allowed maximum Co number is relatively small compared to usually applied conditions for VoF solvers (Windt et al. 2018), potentially increasing the required simulation time.

#### *shallowWaterFoamsrc*

For the *shallowWaterFoamsrc* solver, the grid only needs to be discretised in the horizontal direction, since the free surface elevation is captured by a variable and the vertical grid resolution is fixed to one cell. The horizontal resolution of the grid is expressed in terms of cells per wavelength (CPL). The considered cell sizes are 50, 100, and 200 CPL. The *shallowWaterFoamsrc* solver does not allow the use of variable time stepping and a fixed time step of $\frac{\text{ T }}{100}$ is used. The time traces of the surface elevation, and the convergence characteristics, are plotted in Fig. 11 and listed in Table 4, respectively. Oscillatory converging results are achieved with a grid resolution of 100CPL, resulting in a discretisation uncertainty of $\bar{U}=0.48\%$.

For the temporal convergence study, three different, fixed time step sizes are considered: $\text {d}t=\frac{\text {T}}{100}$, $\frac{\text {T}}{200}$, $\frac{\text {T}}{400}$. The time traces of the surface elevation, and the convergence characteristics, are plotted in Fig. 12 and listed in Table 4, respectively. Monotonically converging results are achieved with a time step size of $\frac{\text {T}}{100}$, resulting in a discretisation uncertainty of $\bar{U}=0.31\%$.

### Numerical beach

Efficient wave absorption in NWTs is important to ensure the replication of open ocean conditions and to minimise the contamination of the wave field with undesired reflected waves. To determined the optimal, i.e. most efficient, settings for the numerical beaches the reflection coefficient, *R*, is considered. Following Mansard and Funke (1980), *R* is calculated following

11 $$\begin{aligned} R = \frac{\hat{S}_{\eta \,\text {R}}}{\hat{S}_{\eta \,\text {I}}}\times 100\%, \end{aligned}$$

where $\hat{S}_{\eta \,\text {I}}$ is the peak value of the spectral density of the incident wave at a frequency $f_{p,\text {I}}$. $\hat{S}_{\eta \,\text {R}}$ is the corresponding spectral density of the reflected wave at $f_{p,\text {I}}$. To separate the incident and reflected wave field, a three point method is proposed by Mansard and Funke (1980), where the free surface elevation time traces are measured at three different wave probes that are spaced at specific relative distances from each other. Based on the guidelines provided by Mansard and Funke (1980), the distance between wave probe 1 and wave probe 2 is set to $\frac{\lambda _p}{10}$, and the distance between wave probe 1 and wave probe 3 is set to $\frac{\lambda _p}{4}$.

In previous studies by the authors (Schmitt et al. 2019; Windt et al. 2019a) it has been shown that, for the VoF type solvers, a numerical beach extending over one wavelength with a damping factor of the order of $\mathcal {O}(10\,\hbox {s})$ can achieve reflection coefficients of less than 5%, which is considered small (Cruz 2007). Informed by these previous studies, the optimal numerical beach settings are determined through a parametric study. Results are listed in Table 5.

**Table 5:** Reflection coefficients for different lengths and damping coefficients of the numerical beach

*interFoamsrc* & *interIsoFoamsrc*				
Damping factor $S_{\text {max}}\,[\hbox {s}^{-1}]$	1.5	3	6	12
Beach length $1\lambda$	3.28	2.52	1.03	1.44

*potentialFreesurfaceFoamsrc*				
Damping factor $S_{\text {max}}\,[\hbox {s}^{-1}]$	5	10	20	40
Beach length $1\lambda$	13.98	6.10	6.54	1.15

*shallowWaterFoamsrc*				
Damping factor $S_{\text {max}}\,[\hbox {m}^{2}\,\hbox {s}^{-1}]$	0.5	1	2	4
Beach length $1\lambda$	3.00	4.52	5.03	11.43

Reflection coefficients of less then 3.5% are achieved with damping factors between $1.5\,\hbox {s}^{-1}$ and $12\,\hbox {s}^{-1}$ for the VoF based solver, which is consistent with the findings by Schmitt et al. (2019); Windt et al. (2019a). The *potentialFreeSurfaceFoamsrc* and *shallowWaterFoamsrc* solvers can be expected to differ significantly in numerical values for the damping term, partly because single phase solvers do not need to consider density, and the shallow water solver solves for depth-averaged impulse.

With the *potentialFreeSurfaceFoamsrc* solver, for a beach length of one wavelength, the smallest reflection coefficient (approx. 1%) is achieved for a damping coefficient of $40\,\hbox {s}^{-1}$. The *shallowWaterFoamsrc* solver also yields reflection coefficients of about 3%, with a damping factor $0.5\,{\hbox {m}^{2}\,\hbox {s}^{1}}$.

It should be noted that, overall, sufficiently small reflection coefficients ($\mathcal {O}(5\%)$) are achieved for all four solvers and results are not very sensitive to the settings, an important feature for application.

### Source shape

As demonstrated by Schmitt et al. (2019), the shape (length and height) of the source region can have a significant influence on the created wave. To determine the optimal source geometry for the different solvers, parametric studies, based on Schmitt et al. (2019), are performed. Here, optimality is defined by a minimal normalised root mean square error (nRMSE) following:

12 $$\begin{aligned} \text {nRMSE}=\frac{1}{H}\cdot \sqrt{\frac{1}{K}\sum ^{K}_{i}\left( \eta _{T,i}-\eta _{R,i}\right) ^2}, \end{aligned}$$

where *H* denotes the target peak-to-trough wave height, *K* is the number of samples, $\eta _{T,i}$ is the target wave and $\eta _{R,i}$ is the resulting wave.

**Fig. 13:**
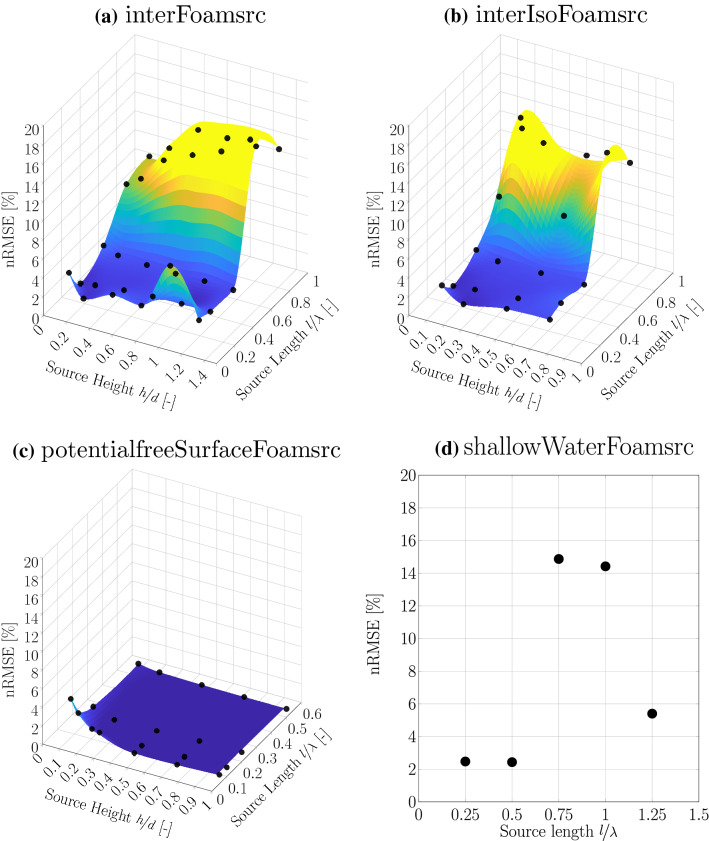
nRMSE values over the source geometry parameter space for the *interFoamsrc*, *interIsoFoamsrc*, *potentialFreeSurfaceFoamsrc*, and *shallowWaterFoamsrc* solver. In the surface plots, the interpolation points are marked in black

For the *interFoamsrc*, *interIsoFoamsrc*, and the *potentialFreeSurfaceFoamsrc* solvers, both, the source length and height have to be optimised. Note that, in the following, the source height, *h*, is parametrised by the water depth, and the source length, *l*, is parametrised by the wavelength. Figure 13a–c show the surface plots of the relative error over the tested parameter space for the *interFoamsrc*, *interIsoFoamsrc*, and the *potentialFreeSurfaceFoamsrc* solvers, respectively. The *shallowWaterFoamsrc* solver is fully defined by the source length. Figure 13d shows the nRMSE values over the tested source lengths.

Characteristic behaviour can be identified for the VoF based solvers, for which a clear drop in error can be observed, for source lengths smaller than $0.5\lambda$. For smaller source lengths, the error plateaus at around 2% for the *interFoamsrc* solver. In the region of source lengths smaller than $0.5\lambda$, only relatively small differences in the error can be observed for different source heights. Overall, minimal errors can be determined for source heights smaller than 0.5*d*. *potentialFreeSurfaceFoamsrc* shows a clear peak in the error for a very small source regions ($l<0.2\lambda$ and $h<0.2d$). However, it should be noted that the maximum error only measures $4.7\%$. For all other configuration, the error plateaus at relatively small values of less then $1\%$. As stated above, the *shallowWaterFoamsrc* solver is fully defined by the source length. For source lengths less than $0.5\lambda$ the nRMSE, is less than $3\%$.

### Results

**Fig. 14:**
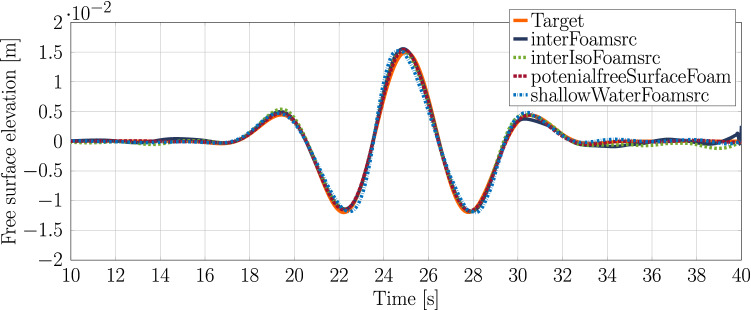
Free surface elevation using the optimised settings for the numerical beach and the source geometry

The measured free surface elevation for each of the solvers, using the optimised settings for the numerical beach and the source geometry, are compared against the target wave signal in Fig. 14. The plot highlights the ability of the solvers to accurately generate the desired wave packet using the impulse wave maker, with a good agreement seen between all of the solvers and the target wave. The nRMSE values are listed in Table 6, where *potentialFreesurfaceFoamsrc* is seen to most closely match the target wave.

**Fig. 15:**
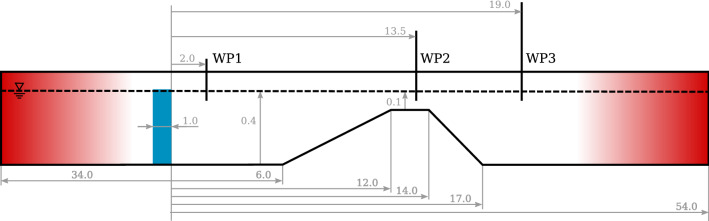
Schematic of the NWT, employed in this case study. The source region is marked in blue. The up-wave and down-wave beaches are marked in red. Additionally the location of the waveprobes (WP) is indicated. Schematic not to scale. All dimensions in [m]

A comprehensive assessment of the computational efficiency of the solvers is beyond the scope of this paper. Schemes and solver settings were not optimised for each application and mesh resolution was chosen to ensure converged results but not optimal efficiency. However, results allow some qualitative indications on computational effort of the methods. As expected, the *shallowWaterFoam* solver is at least an order of magnitude faster than the other solvers which discretise the vertical dimension.

*potentialFreeSurfaceFoamsrc* does not solve for species transport and might be expected to show improvements in computational speed. However, simulation times were found to be similar to the VoF solvers. The requirement of a much lower Co number seems to negate expected efficiency gains. However, in many applications the time step is not limited by wave propagation but motion solvers (Feinberg 2019; Schmitt and Elsäer 2015).

**Fig. 16:**
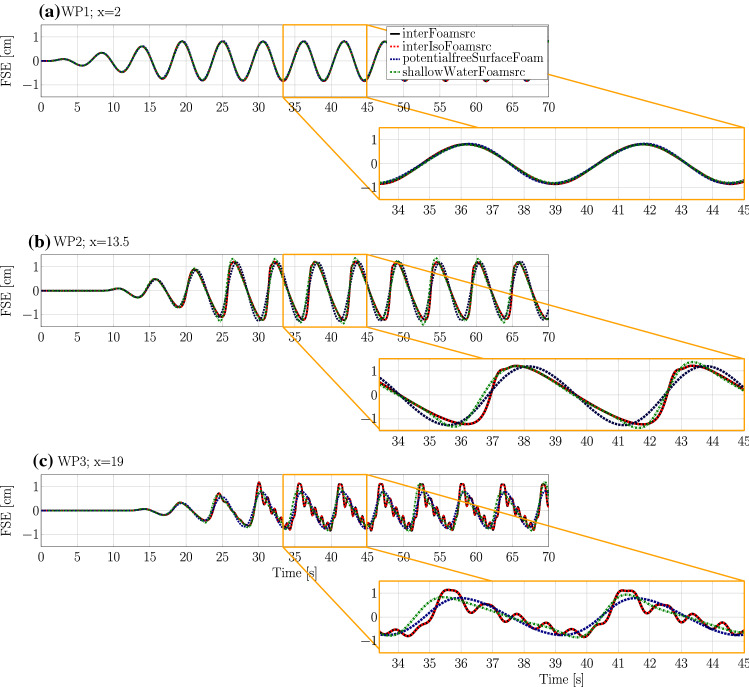
Time traces of the free surface elevation (in [cm]), measured at three different wave probe locations along the NWT, as indicated in Fig. 15. Results are shown for the four considered solvers

## Test case 2: wave propagation over a submerged shoal

This section presents an application of the solvers to wave propagation over a submerged shoal, adapted from the case presented in Dingemans (1994).

**Table 6:** nRMSE values for the optimised settings for the numerical beach and the source geometry

	nRMSE [%]
*interFoamsrc*	1.37
*interIsoFoamsrc*	1.36
*potentialFreesurfaceFoamsrc*	0.70
*shallowWaterFoamsrc*	2.44

- In a first step the interFoamsrc solver was successfully validated against the original experiment in Dingemans (1994). For brevity, the results of the validation study are omitted here, but are provided in the Appendix A. The successful validation then allows the use of interFoamsrc as the reference in the next step.
- In a second step the characteristics of the initial experimental setup are scaled to represent shallow water conditions. This is done to allow a fair demonstration of all the solvers, including *shallowWaterFoam* which is limited to shallow water. The original water depth of $d = 0.4$ m is kept and the shallow water wave characteristics are: $T = 5.6$ s, $H = 0.038$ m, $\lambda = 11$ m, and $\frac{d}{\lambda } = 0.036$.

Figure 15 shows the schematic of the test case, with all relevant dimensions (in [m]). The source region is marked in blue and the up-wave and down-wave beaches are marked in red. The surface elevation is monitored at three different locations, marked as WP1–WP3 in Fig. 15. The mesh discretisation follows the converged parametrisation, in terms of CPH, CPD, and CPL, determined for the previous test case in Sect. 4.1. Using these values as the base discretisation level, a separate convergence study was performed (omitted here for brevity) by doubling and halving the mesh refinement, to confirm that the mesh discretisation is also converged for this test case.

Time traces of free surface elevation, for the four solvers at the three different wave probe locations, are plotted in Fig. 16a–c, respectively. For better visibility, a close up (between 30 and 45 s) is included in Fig. 16. The nRMSE between the surface elevation data at the three wave probes is listed in Table 7, following:

13 $$\begin{aligned} \text {nRMSE}=\frac{1}{\mathcal {N}}\cdot \sqrt{\frac{1}{K}\sum ^{K}_{i}(\eta _\mathrm{interFoamsrc}-\eta _{j})^2}, \end{aligned}$$

where $\eta _\mathrm{interFoamsrc}$ is the surface elevation at a specific wave probe from the *interFoamsrc* solver. $\mathcal {N}$ is the maximum wave height at a specific wave probe from the *interFoamsrc* solver, and $\eta _{j}$ is the surface elevation at a specific wave probe from *interIsoFoamsrc*, *potentialFreeSurfaceFoamsrc*, and *shallowWaterFoamsrc* solver.

**Table 7:** nRMSE values for the different solvers compared to the *interIsoFoamsrc* reference values

nRMSE (%)	WP1	WP2	WP3
*interIsoFoamsrc*	0.13	0.08	0.09
*potentialFreeSurfaceFoamsrc*	1.80	8.43	10.10
*shallowWaterFoamsrc*	1.20	6.41	10.04

From Fig. 16, different levels of agreement between the solvers can be observed at different wave probe locations. At WP1, closest to the source region and furthest away from the shoal, the measured free surface elevations from the different solvers virtually overlay each other. This indicates that, for undisturbed wave propagation, all solvers perform equally well.

On top of the shoal, at WP2, major deviations between the solvers are visible. While *interFoamsrc* and *interIsoFoamsrc* deliver consistent results, *shallowWaterFoamsrc* and *potentialFreeSurfaceFoamsrc* are not able to capture the deformation of the wave shape caused by wave-structure interaction. From the close-up in Fig. 16b, it can be seen that the *shallowWaterFoamsrc* solver agrees slightly better with the VoF solvers, compared to *potentialFreeSurfaceFoamsrc*, capturing the surface elevation well for the second part of the wave period, after the wave crest. The results from *potentialFreeSurfaceFoamsrc* seems less affected by the shoal, resulting in an almost linear wave shape. Regardless of the differences in the shape of the waves at WP2, the wave amplitude is in reasonable agreement for all the solvers.

**Fig. 17:**
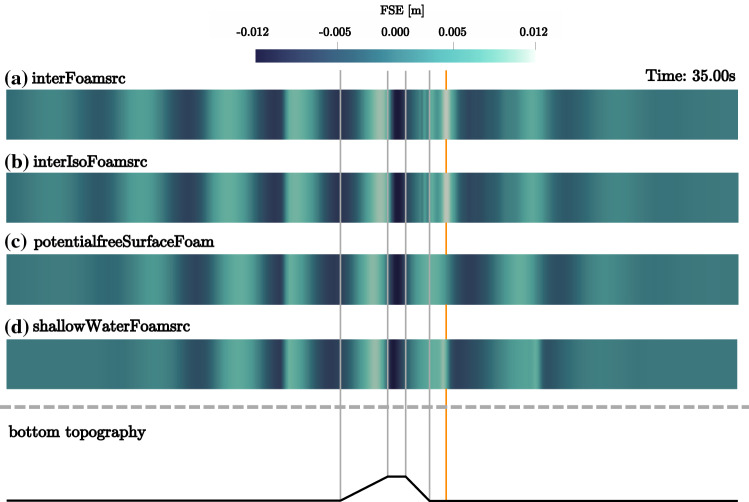
Screen shots (top view) of the free surface elevation (in [m]) for the four different solvers at time $t=35\,\hbox {m}$. For reference, the figure includes a schematic of the bottom topography. The main peak after the shoal is marked in orange

Behind the shoal, at WP3, the largest differences between the solvers can be observed. Again, *interFoamsrc* and *interIsoFoamsrc* deliver consistent results, showing high-frequency ripples induced by the wave-structure interaction. These ripples are neither captured by *shallowWaterFoamsrc* nor *potentialFreeSurfaceFoamsrc*. Similar to the findings at WP2, slightly better performance can be observed for *shallowWaterFoamsrc*. *potentialFreeSurfaceFoamsrc* yields an almost linear wave shape. *shallowWaterFoamsrc* and *potentialFreeSurfaceFoamsrc* are both not able to capture the wave amplitude correctly.

Wave dissipation is a well-documented issue in the application of VoF solvers. While this paper does not specifically investigate this issue, results do not show a major decline in wave height along the wave tank for any of the alternative solvers.

Screenshots of the wave field in the numerical domain at a specific time instance ($t=35$ s) are shown in Fig. 17a–d for *interFoamsrc*, *interIsoFoamsrc*, *potentialFreeSurfaceFoamsrc*, and *shallowWaterFoamsrc*, respectively. Note that the screen shots show a top–down view on the wave field. The numerical domains have been stretched in the lateral direction for better visibility.

The screenshots underline the findings from the free surface elevation time traces in Fig. 16a–c. While the amplitude of the free surface elevation just above the shoal is in relatively close agreement for all the solvers, *potentialFreeSurfaceFoamsrc* and *shallowWaterFoamsrc* show considerable differences, specifically in the wave field after the shoal, compared to *interFoamsrc* and *interIsoFoamsrc*. *shallowWaterFoamsrc* is able to capture the main peak after the shoal (marked in orange in Fig. 17), with some degradation in wave height; however, after this main peak, the wave fields do not agree. *potentialFreeSurfaceFoamsrc* is generally not able to capture the transformation of the wave field after the shoal.

**Fig. 18:**
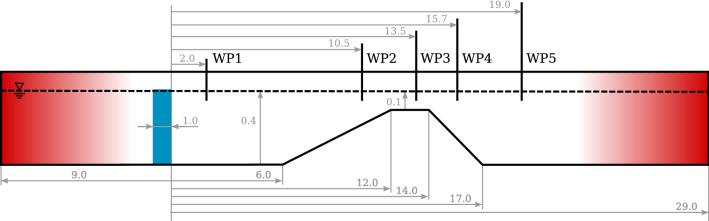
Schematic of the NWT, employed for the validation study of *interFoamsrc*. The source region is marked in blue. The up-wave and down-wave beaches are marked in red. Additionally the location of the wave probes (WP) is indicated. Schematic not to scale. All dimensions in [m]

**Fig. 19:**
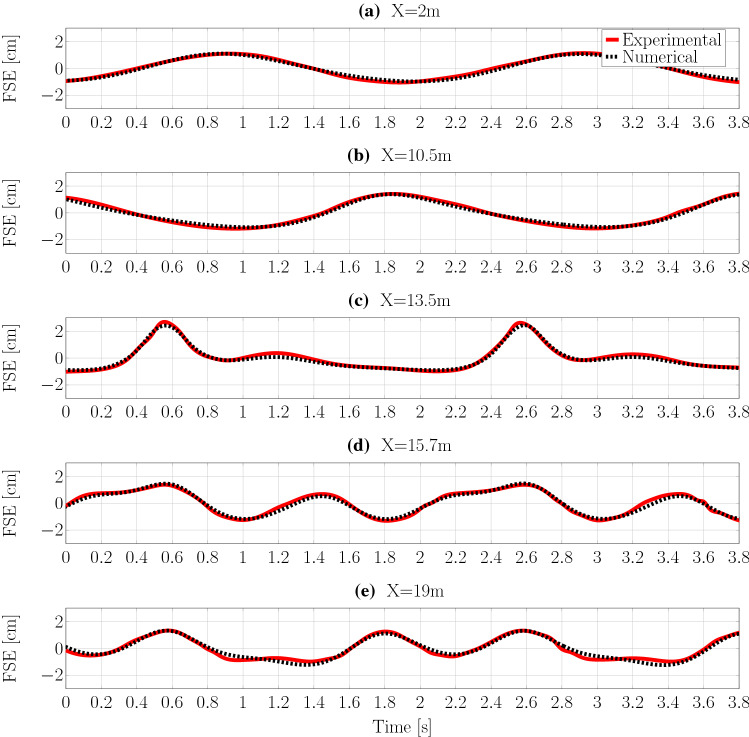
Experimental and numerical free surface elevation data at five different locations along the wave tank

## Conclusions

This paper presents the inclusion of wavemaking and absorption capabilities to four OpenFOAM solvers based on different conceptual models: surface tracking (*potentialFreeSurfaceFoam*), shallow water equations (*shallowWaterFoam*) and the conventional VoF (*interFoam*, *interIsoFoam*). All solvers are demonstrated to accurately reproduce a multi-frequency wave packet progressing over constant bathymetry with negligible reflection. Changes in wave shape and ripple waves, created by a wave passing over a submerged shoal, are only captured in full detail by VoF based methods. As expected the 2-D solver *shallowWaterFoam* requires orders of magnitudes less mesh cells and computing time than the other methods. The inclusion of NWT functionality to a wider range of solvers is hoped to foster discussion and developments beyond the already well-established VoF methods.

Alternative methods beyond the already well established VoF methods are worth considering because potential for significant reductions in computational effort exist, opening up new areas for the application of *OpenFOAM* to ocean engineering problems.

## Appendix A: Validation of *interFoamsrc*

To ensure the correct solution of the wave-structure interaction problem, a validation study for the *interFoamsrc* solver is performed, based on the case study of the wave propagation over a submerged shoal, using the experimental data presented by Dingemans (1994). Figure 18 shows the schematic of the NWT, used in the validation study, with all relevant dimensions (in [m]). The source region is marked in blue and the up-wave and down-wave beaches are marked in red. The surface elevation is monitored at five different locations, marked as WP1–WP5 in Fig. 18. The wave characteristics are: $T=2.2\,\hbox {s}$, $H=0.02\,\hbox {m}$, $\lambda =4.11\,\hbox {m}$, and $\frac{d}{\lambda }=0.097$.

Figure 19a–e show the time traces of the experimental and numerical free surface elevation, extracted from WP1–WP5, respectively. Overall, close agreement between the experimental and numerical results can be observed. Specifically, on top of the shoal (WP3), as well as after the shoal (WP4 and WP5), *interFoamsrc* proves to the able to capture the non linear wave field, induced by the WSI. With these results, *interFoamsrc* can be considered as validated for the specific wave–structure interaction problem of wave propagation over a submerged shoal, and considered as reference for the case study presented in Sect. 5.

## Footnotes

1 Variations of the time stepping are applied according to the time discretization used
